# The advantages of NK cell vaccines in solid carcinoma clinical trials: conducted by various biology strategy and technology

**DOI:** 10.3389/fmed.2025.1656570

**Published:** 2025-10-17

**Authors:** Yizhe Hu, Limei Leng, Bing Li, Qiang Qiao

**Affiliations:** Ansteel Group General Hospital, Anshan, China

**Keywords:** natural killer (NK) cell-based vaccines, solid tumors, clinical trials, tumor microenvironment, cancer immunotherapy

## Abstract

This review systematically discusses the latest clinical progress and challenges of natural killer (NK) cell vaccines in the treatment of solid tumors. By searching databases such as ClinicalTrials.gov and PubMed (2019–2025), we focused on preclinical studies and Phase I/II/III registration trials in the past 2–3 years to dissect the mechanism of action and efficacy data of different vaccine platforms. The study illustrated: Dendritic cell-based vaccine platforms (e.g., ilixadencel), cytokine-based vaccine platforms (e.g., ALT-803), NK receptor agonist antibodies (e.g., AFM24) and mRNA/LNP-based vaccine platforms (e.g., BNT116) It has shown early efficacy in solid tumors such as non-small cell lung cancer, triple-negative breast cancer, and glioblastoma (with partial ORR of 30–50% and DCR of 80–100%), and the safety is comparatively manageable (the incidence of grade ≥3 adverse events is less compared to T-cell therapy). However, complex manufacturing procedures, inhibition of the tumor microenvironment, and low targeted delivery efficiency remain the main obstacles to transformation. In the future, combinatorial regimens (e.g., sequential application of PD-1 inhibitors) need to be optimized, an iPSC-NK universal platform developed, and perioperative application scenarios explored. NK vaccines, by reshaping the immune microenvironment, will be an attractive strategy to break the bottlenecks in the treatment of solid tumors.

## Graphical abstract

**Figure:**
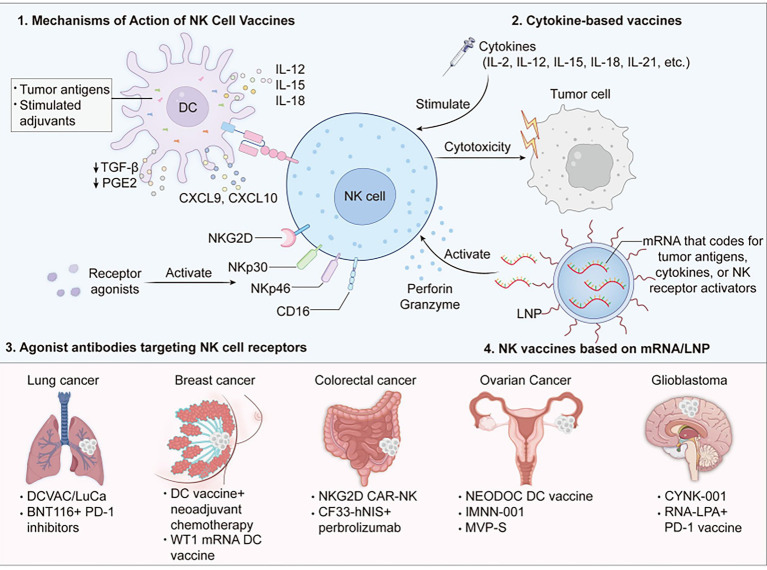

## 1 Introduction

Immunotherapy of solid tumors faces several challenges. The initial challenge is immunosuppression by the tumor microenvironment (TME): Components such as cancer-associated fibroblasts (CAFs), regulatory T cells (Tregs), and myeloid-derived suppressor cells (MDSCs) inhibit T cell and NK cell infiltration and activity by secreting factors such as TGF-*β* and IL-10 (1). When TGF-β binds to the surface receptors of NK cells, SMAD2/3 is phosphorylated. The phosphorylated SMAD2/3 forms a complex with SMAD4 and moves into the nucleus, which can inhibit the expression of proliferation-related genes and cytotoxicity-related genes in NK cells, thereby reducing the cytotoxicity of NK cells (2). Meanwhile, tumor heterogeneity (as manifested by differences in immune cell spatial distribution, antigen loss via clonal evolution) and defects in antigen presentation (e.g., MHC-I down-regulation) still hinder the efficacy of T-cell therapy (3). Clinical trials demonstrate that PD-1/CTLA-4 inhibitors have low response rates in solid tumors such as pancreatic cancer and glioblastoma (4), and CAR-T cells are not able to initiate long-lasting anti-tumor activities due to problems such as poor homing and suppression by the TME.

In such conditions, Natural killer cells (NK cells) overcome the limitations of T cell-based therapies and demonstrate unique anti-tumor value. NK cells can directly destroy tumor cells and virus-infected cells without previous activation. It effectively supplements the blind spot of T-cell therapy by targeting the tumor cells with low expression of MHC-I through the “missing self” mechanism. This characteristic endows NK cells with a selective killing advantage for drug-resistant tumors with MHC-I down-regulation through immune editing (5). The functionality of NK cells is intrinsically linked to the immune microenvironment, wherein numerous factors can exert influence, such as genomic stability. Genomic stability serves as a pivotal determinant in maintaining genomic integrity, and its alterations may significantly impact the activation and functional capacity of NK cells (6, 7). Furthermore, the infiltration ratio of immune cells also affects the immune function of NK cells, such as macrophages and CD8 + T lymphocytes. Macrophages influence the activity of NK cells through the secretion of cytokines and direct phagocytosis (8, 9), while CD8 + T lymphocytes work in synergy with NK cells to enhance the anti-tumor immune response and jointly regulate the immune response in the tumor microenvironment (10). Based on these biological characteristics, NK cell therapy has been one of the key strategies to break through the predicament of solid tumor treatment in recent years, and it mainly includes adoptive NK cell infusion and NK cell vaccines. Adoptive NK cell infusion directly exerts its anti-tumor function through the expansion of NK cells or genetic modification *in vitro* and reinfusion into patients, but its activity is hampered by the short survival time of the cells *in vivo* and immunosuppression by the TME. In contrast, NK cell vaccines aim to stimulate, expand, or guide the patient’s own NK cells in vivo through an active immune strategy, which not only enhances the tumor-targeting ability of NK cells but also potentially induces long-term immune memory, thereby destroying the inhibitory shield of the TME. The NK cell vaccine has demonstrated unique advantages in clinical trials for solid tumors, offering good tolerance, strong anti-tumor activity and flexible treatment strategies, bringing new hope to patients (11).

NK vaccines function through four basic mechanisms, including: (1) Indirect stimulation of NK cells by presenting tumor antigens and adjuvants through antigen-presenting cells (APCs); (2) Local or systemic administration of specific immune-stimulating cytokines to create a highly activated microenvironment of immunity to stimulate NK cells; (3) Direct targeting of the activation receptors on NK cells (e.g., NKG2D, CD16) to enhance their killing function; (4) By delivering mRNA for tumor antigens, cytokines or NK receptor agonists encapsulated in lipid nanoparticles (LNP) to cells and expressing the target molecules, the NK cells are stimulated and their killing function is enhanced. These strategies represent a new breakthrough direction for the treatment of solid tumors and can be expected to exert synergistic effect in combination with other immunotherapies, i.e., PD-1 inhibitors. This review aims to give a systematic update on the latest progress of NK vaccines for treating solid tumors, with emphasis on the analysis of clinical trial platforms, data, challenges for clinical translation, and prospects.

## 2 Mechanisms of action of natural killer (NK) cell vaccines

### 2.1 NK vaccines based on dendritic cells (DC)

Dendritic Cells (DC), as professional antigen-presenting cells, play important roles in linking innate immunity and adaptive immunity. Dc-based NK vaccines, with loaded tumor antigens and stimulated adjuvants, mature and reinfused into the patient, activate NK cells directly and recruit them to move into the tumor microenvironment (TME), and also create long-term immune memory (12), having the ability for synergy and enhancement of effectiveness (13). The major mechanisms are: mature DCS secrete IL-12, IL-15, IL-18, which directly induce NK cell proliferation and killing; DC surface antigens engage with NK receptors to provide co-stimulatory signals; DCS secrete CXCL9/CXCL10 to recruit NK cells (14) and inhibit inhibitors such as TGF-*β* and PGE2. Based on these mechanisms, several DC-NK vaccines are in the clinical trial phase for solid tumors.

Ilixadencel, which is derived from healthy donor cells, has the ability to rapidly stimulate the patient’s native DC cells upon injection into the tumor and thus induce an anti-cancer immune response (15–17). A Phase II clinical trial called MERECA (NCT02432846) proved that in patients with advanced renal cell carcinoma (mRCC), Ilixadencel combined with the anti-cancer drug sunitinib was better than sunitinib alone: More people had reduced tumor size (higher than doubling overall response rate), more people had complete disappearance of the tumor (higher complete response rate), and the effect lasted longer (18).

DCVax-L would generally load its own DC cells using the patient’s own tumor fragments (lysates). DCVax-L will potentially initiate the polyclonal T cell response, indirectly boost NK cell function (by cross-activation via cytokines like IFN-*γ*), and obtain a broad-spectrum anti-tumor immunity, hailing new hope for glioblastoma treatment. The most recent Phase III clinical trial (NCT00045968) indicates that DCVax-L can successfully extend the survival time of glioblastoma patients and has favorable safety (19).

### 2.2 Cytokine-based vaccines

Cytokine vaccines achieve their antitumor activity against solid cancers via induction of a strongly immunostimulatory milieu *in vivo* following the local or systemic administration of single cytokines (and their combinations, fusion proteins, sustained-release formulations), straightaway augmenting the growth, activation, and cytotoxicity enhancement of the endogenous NK cells and their trafficking and infiltration into tumor tissues. Amongst these common cytokines are IL-2, IL-12, IL-15, IL-18, IL-21, etc. (20), and some related clinical trials are already evaluating their possibility of use. Alt-803, for example, is an IL-15 analogue that induces activation, expansion, and growth of NK and CD8+ + T cells. In the first Phase I human trial (NCT01885897), 19 percent of blood cancer patients were found to enjoy clinical benefit after their first dose of ALT-803 (21).

### 2.3 Agonist antibodies targeting NK cell receptors

Agonist antibodies augment the cytotoxic activity of NK cells directly or exert cytotoxic activity against tumors by binding specifically to activated surface receptors of NK cells (e.g., NKG2D, CD16, NKp30, NKp46). Agonist antibodies are divided into two classes based on design strategy: single-target agonist antibodies and bispecific/trispecific antibodies, promoting anti-tumor efficacy by activating a single NK receptor pathway. Prototypic targets are NKG2D receptor agonists, CD16 (FcγRIIIa) agonists, and NKp30/NKp46 agonists. Bispecific/trispecific antibodies enable tumor-specific recruitment and activation of NK cells by interacting with tumor-associated antigens (TAA) on one side and NK-activating receptors (CD16, NKp30, etc.) on the other side.

DF1001 is a trispecific antibody targeting NKG2D, HER2 (cancer marker), and CD16a simultaneously. Targeting HER2 as an anchor to modulate adaptive and innate immunity can redirect NK and CD8 T cells to target tumors, reshaping the tumor microenvironment and making “cold” tumors “hot” tumors (NCT04143711) (22).

Another new bispecific EGFR/CD16A bispecific antibody, AFM24, kills tumor cells directly by activating NK cells and macrophages. Good tolerance and early anti-tumor activity (NCT04259450) were demonstrated in Phase I trials in patients with advanced solid tumors expressing epidermal growth factor receptor (EGFR) (23).

SAR443579 is a dual-specific antibody against the CD123 antigen, which simultaneously binds to NKp46 and CD16a on NK cells, enabling cytotoxic synapse establishment between NK cells and CD123-expressing tumor cells. NK cell activation and tumor killing were well-tolerated in phase 1/2 trials (NCT05086315) in r/r AML, B-cell acute lymphoblastic leukemia, or high-risk myeloproliferative neoplasms patients, and clinical activity was observed (24).

### 2.4 NK vaccines based on mRNA/LNP

NK vaccines that use mRNA and lipid nanoparticles (LNP) are designed by encapsulating mRNA that codes for tumor antigens, cytokines, or NK receptor activators within lipid nanoparticles. These tiny lipid particles protect the mRNA from breakdown by enzymes in our bodies, help it stay stable, and make sure it gets into cells effectively. Once inside, the mRNA instructs the cells to produce specific molecules that activate natural killer (NK) cells and boost their ability to attack tumors. For example, a special imidazole-based lipid platform can deliver IL-2 mRNA to NK cells with very low toxicity, which could lead to better cancer treatments (25). Also, GSDMBNT mRNA@LNPs can attract NK cells to the tumor area. When activated, these NK cells release toxic substances like perforin and granzyme, directly killing cancer cells (26). This kind of lipid nanoparticle shows a lot of promise for future clinical use (Table 1).

**Table 1:** Overview of NK cell vaccine platforms, their mechanisms, advantages, and challenges.

Vaccine platfom	Core mechanism	Main advantages	Main disadvantage/Challenges	Representative product/Companies	Cinical trial phase (NCT Number)	Targeted solid tumor types
Dendritic cell (DC-based)	Mature DCs loaded with tumor antigens: Secrete IL-12/IL-15/L-18 to promote NK proliferation and cytotoxicity Provide co-stimulatory signals via surface molecules Secrete CXCL9/CXCL10 to recruit NK cells to TME	Activates innate and adaptive immunity Induces long-term Immune memory Clinically validated survival benefit	High cost and lengthy preparation Complex personalized manufacturing TME immunosuppression may limit efficacy	Ilixadencel (allogeneic DC) DCVax-L(autologous DC)	Ilixadencel:Phase II(NCT02432846) DCVax-L:Phase III(NCT00045968)	Metastatic Renal Cell Carcinoma(mRCC) Glioblastoma
Cytokine-based	Administration of cytokines (IL-2,IL-12,IL-15,IL-18,IL-21): Directly promote NK expansion and activation Enhance cytotoxic function Recruit NK cells to tumor sites	Direct action, rapid effect Systemic or local administration High potential for combination therapies	Systemic toxicity (e.g., cytokine release syndrome) Short half-life requiring frequent dosing May activate regulatory immune cells	ALT-803(IL-15 mimetic)	Phase l (NCT01885897)	Hematological malignancies°(solid tumor potential)
Agonist antibodies targeting NK cell receptors	Single-target antibodies: Activate NK receptors(NKG2D,CD16,NKp30,NKp46) Bi/tri-specific antibodies: Bind tumor antigens with one arm and NK receptors with the other	Precise tumor targeting Bi/tri-specifics redirect immune cells Convert "cold tumors to hot"	Tumor antigen heterogeneity may cause escape Risk of off-target toxicity Requires affinity optimization	DF10D1 (HER2/NKG2D/CD16) AFM24(EGFR/CD16A) SAR443579 (CD123/NKp46/CD16)	DF1001:PhaseI(NCT04143711) AFM24:PhaseI(NCT04259450) SAR443579:Phase I/II(NCT05086315)	Advanced solid tumors(e.g.HER2+/EGFR+) Hematological tumors(SAR443579)
mRNA/LNP-based	LNP-encapsulated mRNA encoding tumor antigens/cytokines/NK receptor agonists: Delivers genetic instructions to cells Activates NK cells Enhances cytotoxic function	Rapid and flexible antigen design LNP protects mRNA and enables efficient delivery Potential for engineering NK cells	In vivo delivery efficiency needs optimization LNPs may cause inflammatory responses Limited long-term safety data	IL-2mRNA@imidazole lipid platform GSDMBNT mRNA@LNPs	Preclinical studies(No NCT listed)	Broad solid tumor potentlal

## 3 Focus on ongoing clinical trials: indications and preliminary results

### 3.1 Organize the discussion by solid tumor type

#### 3.1.1 Lung cancer (NSCLC, SCLC)

Lung cancer is one of the most common and deadliest cancers worldwide. While traditional treatments (surgery, chemotherapy, radiotherapy) can prolong life, they have their own drawbacks, such as surgery cannot completely eliminate the tumor, chemotherapy has significant side effects, and radiotherapy can also harm normal tissue. In the last couple of years, the introduction of immunotherapy has brought a ray of hope in the management of lung cancer. NK cell immunotherapy is gradually moving into the limelight of research. DCVAC/LuCa is an autologous dendritic cell-loaded lung cancer cell lysis product for first-line treatment of stage IV non-squamous non-small cell lung cancer (NSCLC) (NCT02669719) with ORR 31.82% and median PFS of 8.0 months (27). BNT116 is an LNP that delivers mRNA for six antigens of lung cancer (NY-ESO-1, MAGE-C2, etc.), activates antigen-specific T cells, and indirectly recruits/activates NK cells through the inflammatory microenvironment. Combined with the PD-1 inhibitor simiprimab in PD-L1-positive advanced NSCLC patients (who had previously received ≤2 lines of therapy), ORR was 10%, DCR was 80%, and median PFS was 5.5 months (NCT05142189) (28). In the phase 2b/3 trial of TG4010 vaccine, vaccine activity was more apparent in patients with non-squamous tumors and low TrPAL. In patients with non-squamous tumors, the TG4010 arm increased ORR by 9.7% and PFS by 1.8 months; In patients with low TrPAL, PFS was prolonged by 2.3 months (NCT01383148) (29).

#### 3.1.2 Breast cancer (TNBC, HER2+, etc.)

Current breast cancer clinical trials of NK cell vaccines focus mainly on three strategies: cytokine adjuvant vaccines, oncolytic virus-NK combination therapy, and NK receptor agonist antibodies. In patients with initial HER2-negative breast cancer, adding a dendritic cell vaccine to neoadjuvant chemotherapy (preoperative chemotherapy) (NCT01431196) significantly increased the percentage of pathological complete response (pCR, or disappearance of the tumor after surgery) to 26.3% and reduced tumor stage (surgical downstaging) in 13% of patients (30). Another dendritic cell vaccine with WT1 protein mRNA (NCT01291420) showed striking tumor reduction (ORR) in 50% of 40 hormone receptor-negative/HER2-negative metastatic breast cancer (HR−/HER2 − MBC) patients at 6 months posttreatment. Condition was controlled in all the patients (DCR 100%) in this single-arm study (31).

#### 3.1.3 Colorectal cancer

In the context of colorectal cancer, NK cell-targeted therapies have shown multi-dimensional advancement: CAR-NK cells targeting NKG2D (NCT05213195) noted 100% control of disease following local infusion (32); CF33-hNIS oncolytic virus monotherapy or pembrolizumab combination considerably increased NK activity, achieving an 86% DCR (32, 33); While the CEA-DC vaccine indirectly activates NK cells, a Phase II clinical trial for a dendritic cell vaccine in MSI-type CRC is underway, but currently has good safety and tolerability (NCT01885702) (34). Despite the striking long-term anti-relapse effectiveness of autologous NK infusion (100% RFS at 3 years), its ‘vaccine’ status also needs to be established further.

#### 3.1.4 Ovarian cancer

Several technical avenues of NK cell vaccine strategies in ovarian cancer. NEODOC dendritic cell vaccine (NCT05773859) demonstrates good initial safety and induces a strong immune response by immunizing autologous tumor antigens with chemotherapy (35); Cytokine vector IMNN-001 significantly prolonged OS to 40.5 months in the Phase II study with first-line chemotherapy because of the IL-12-induced NK/T cell activation effect (35% risk reduction of death vs. control, HR = 0.65) (NCT03393884) (36). In platinum-resistant patients, MVP-S polypeptide vaccine combined with the immunomodulator cyclophosphamide achieved a 63% disease control rate (NCT02785250) (37). Novel mRNA vaccines (e.g., mCM10-L against MICA/B) and viral adjuvant vaccines have been demonstrated in preclinical models to synergize with PD-1 inhibitors to enable NK cell killing activity, which requires confirmation in clinical translation as a high priority (38).

#### 3.1.5 Glioblastoma

Current clinical trials of NK vaccines in GBM are based chiefly on cell infusion (e.g., CYNK-001 NCT04489420) (39) and antigen delivery platforms (e.g., RNA-LPA, GlioVax (40)), which first establish safety and immune activation capability; PD-1 inhibitors in combination can significantly improve the ORR (e.g., RNA-LPA by 50%), but the long-term PFS benefits are yet to be established with expansion of sample size. Of interest is the local delivery method used (lumbar puncture/Ommaya capsule) may maximize tumor microenvironment infiltration as a new avenue toward bridging the immunosuppressive barrier.

#### 3.1.6 Solid cancers other than mentioned (pancreatic cancer, liver cancer, gastric cancer, melanoma, etc.)

Current clinical research involving NK vaccines in solid tumors primarily focuses on the combination with immune checkpoint inhibitors to bypass tumor microenvironment suppression. In cancer of the liver, mRNA vaccine GNOS-PV02 combined with PD-1 antibody yielded an ORR of 30.6% (NCT04251117) (41); Pancreatic cancer neoantigen vaccine BNT122 profoundly prolonged recurrence-free survival (HR = 0.08) (42). Although efficacy for gastric cancer and melanoma is yet to come, the early trials showed that the safety of related therapies (e.g., EVM16 without severe dose-limiting toxicity) is manageable. New vectors, including bacterially based vaccines, have been promising to be more effective in laboratory models.

### 3.2 Combination therapy approach

NK vaccines reprogram the immune microenvironment of tumors (TME) through the activation of endogenous NK cell activity. While NK cell vaccines show promise for the treatment of solid tumors, the therapeutic effect is limited in monotherapy, but combining with other treatments can significantly enhance the anti-tumor activity. The major approaches are the use of immune checkpoint inhibitors (PD-1/PD-L1) in combination. The highly expressed PD-L1 in the tumor microenvironment can inhibit the cytotoxicity of NK cells (through the PD-1/NKp30 signal). Promoting the upregulation of NKGZD expression on the surface of NK cells significantly increases the secretion level of IFN-*γ* in tumor tissues, thereby continuously enhancing the efficacy of tumor immunotherapy. ICI blocks the PD-1/PD-L1 pathway to prevent NK cell inhibition and enhance vaccine-induced NK cell activation and infiltration (43). The Ib/II phase study data of Nectin-4 ADC 9 MW2821 combined with PD-1 antibody Toripalimab in patients with locally advanced or metastatic urothelial carcinoma (la/mUC) showed an ORR of 80%; The disease control rate (DCR) was 92.5% (NCT06079112) (44), and the objective response rate (ORR) was 83%. Clinical trial data on AFM13 and pembrolizumab combination yielded an objective response rate (ORR) of 83% (NCT02665650) in relapsed/refractory Hodgkin’s lymphoma (45). Additionally, NK cell vaccines with chemotherapy or radiotherapy induce danger signals and antigens by inducing immunogenic cell death, killing immune-suppressive cells, increasing the tumor microenvironment, and potentially increasing the sensitivity of tumor cells to NK cell killing. This strategy turns cytotoxic therapy into an “*in situ* vaccine,” repolarizing the immunosuppressive TME into a pro-immunity microenvironment, significantly enhancing NK cell recruitment, survival, and killing function (e.g., NKG2D-L DNA vaccine + TMZ + radiotherapy NCT04290858 (46, 47)). As far as combined targeted therapy is concerned, modulation of antibody-dependent cytotoxicity (ADCC) effect (NK cells with target antibodies) or direct bridging and activation of NK cells by bispecific antibodies/agonists is the main mechanism for tumor cell killing. Combined targeted antibodies (e.g., anti-EGFR /HER2) can greatly enhance NK cell cytotoxicity for tumors (e.g., SNK01 + AFM24, NCT05099549 (48)). Some targeted agents (e.g., HDAC inhibitors) can upregulate the expression of the activating ligand of tumor NK cells. Bispecific antibodies/adaptors can specifically connect NK cells to cancer cells, highly activate and redirect NK cells [e.g., AFM24 + allogeneic NK cells (49, 50)]. In addition to the above therapies, other immunotherapies can be combined, such as combined cell therapy (MUC1-NK/T vaccine NCT04011033) in pancreatic cancer (51); The oncolytic viruses (OV) could be combined with direct lysis of cancer cells to release antigens and be equipped with immune-stimulating factors (such as IL-15/IL-18), initiating a cascade activation effect with the NK vaccine. Activated NK cells can directly lyse OV-infected cancer cells and modulate antiviral immunity (52) (Table 2).

**Table 2:** Selected ongoing clinical trials of NK cell-based therapies in solid tumors.

NCT number	Title	Phase	NK Platform/Product	Target solid tumor(s)	Treatment regimen	Primary endpoint(s)	Estimated completion	Status and preliminary results
NCT06079112	Phase Ib/II Study of 9MW2B21(Nectin-4 ADC)+Toripalimab in la/mUC	Ib/Il	Non-NK vaccine (Combination)	Locally advanced/metastatic urothefial carcinoma (la/mUC)	9MW2821(Nectin-4 ADC) +Toripalimab(PD-1 antibody)	ORR, DCR	Pending	Active; ORR80%, DCR92.5% (35)
NCT02665650	AFM13+ Pembrolizumab for R/R Hodgkin Lymphoma	I/I	Bispecific NK engager(AFM13)	Relapsed/refractory Hodgkin lymphoma	AFM13(CD30/CD16A bispecific antibody)+ Pembrolizumab(PD-1 antibody)	ORR, Safety	Dec 2026	Active; ORR83% (36)
NCT04290858	NKG2D-L DNA Vaccine + Temozolomide+ Radiotherapy for Newly Diagnosed Glioblastoma	I/I	NKG2D ligand DNA vaccine	Glioblastoma	NKG2D-L DNA vaccine Temozolomide(TMZ)+ Radiotherapy	Safety, immune response, PFS	Jun 2025	Recruiting; Preclinical synergy demonstrated (37, 38)
NCT05099549	SNK01(Autologous NK Cells)+AFM24 (EGFR Bispecific Antibody)for Advanced Solid Tumors	I	Autologous NK cells(SNK01)	EGFR+soid tumors (e.g., colorectal cancer)	SNK01+AFM24 (EGFR/CD16A-targeting bispecific antibody)	Safety, MTD	Dec 2024	Active; Preclinical ADCC enhancement (39)
NCT04011033	MUC1-Targeted NK/T Cell Vaccine for Pancreatic Ductal Adenocarcinoma	I	MUC1-CARNK/T cell vaccine	Pancreatic cancer	MUC1-CAR-NK/T vaccine +Chemotherapy(Nab-paclitaxel/Gemcitabine)	Safety, Immune response	Mar 2025	Recruiting: Mechanistic synergy shown (42)
NCT04390399	ANKTIVA (IL-15 Superagonist) + PD L1 t-haNK for Metastatic Pancreatic Cancer (QUILT-88)	lI	CAR-NK(PD-L1 t-haNK)	Metastatic pancreatic cancer	ANKTIVA+PDL1 t–haNK +Standard chemotherapy	OS, Lymphopenia reversal	Jun 2026	Active; RMAT designation (3, 40)
NCT03228667	PD-L1 t-haNK+NK Cell Activator for NSCLC (QUILT-3.055)	II	CAR-NK (PD-L1 t-haNK)	Non-small cell lung cancer (NSCLC)	PD-L1 t-haNK+ANKTIVA + Checkpoint inhibitors	ORR, Safety	Dec 2025	Active; Preliminary survival benefit (3, 41)

### 3.3 Safety overview

NK cell vaccines (e.g., autologous/allogeneic NK adoptive therapy, CAR-NK, etc.) will be tolerable in solid tumor therapy by 2025. Most of the treatment schemes are marked with low-grade adverse events (TRAEs) and have much fewer grade ≥3 events than those with T-cell therapy. Typical TRAEs are injection site reactions (pain, redness, swelling), flu-like syndrome (transient fever, fatigue), and minor abnormalities in liver function tests, which can be treated symptomatically to a large extent. Some SAEs and some toxicities are as follows: The incidence of cytokine release syndrome (CRS) is lower than that of CAR-T therapy, and it is also less severe (≤ grade 2). Studies reported that CRS in allogeneic CAR-T therapy of hematologic malignancies happened in 5–10%, while in solid tumors, it was lower. Closely observe IL-6 and C-reactive protein, and treat with tocilizumab when necessary. There are no certain reports of NK cell therapy resulting in classic ICANS presentations (i.e., epilepsy, brain edema). In studies targeting children, the incidence of ICANS with CAR-NK was significantly lower than with CAR-T (incidence <1% vs. 44%), and this might be explained by the fact that the pathway of activation in NK cells is not dependent on the T cell pathway (53). Off-target toxicity occasionally occurs in EGFR/HER2 CAR-NK therapy as reversible lung injury or liver enzyme elevation, which can be prevented by gene editing for knockout of inhibitory receptors (e.g., NKG2A) or target affinity modification. The incidence of grade ≥3 immune-related adverse events (irAEs) is about 4 to 10%, ranging from immune pneumonia to hypothyroidism and hematotoxicity (e.g., thrombocytopenia). Be vigilant of asynchronous onset of more than one system irAEs (63% of patients do not present simultaneously), and screening at baseline and dynamic monitoring of organ function for autoantibodies is recommended (54).

Present day clinical management strategies are: preinfusion with antihistamines/antipyretic analgesics and preventive measures to avoid symptoms of influenza; typing of NK cells that are allogeneic for HLA to reduce the risk of GVHD (55): Graded treatment, 1–2 grade irAEs managed with local/oral glucocorticoids, ≥3 grade requires stopping the treatment and initiating methylprednisolone shock (1–2 mg/kg/d): There is no clear report of resistance at present, but the problem of decreased persistence of effector cells upon repeated infusions must be watched out for. The 3-year follow-up results showed that autologous NK cells were not linked to delayed toxicity, and the quality of life score did not change (56). In general, NK cell vaccine safety can be controlled, but the synergistic risk of toxicity due to combination regimens (e.g., PD-1 inhibitors) is yet to be established by expanding the sample size. It is recommended to establish specific toxicity grading criteria for solid tumors (such as the modified ASTCT scale) and further enhance safety through maximal optimization of CAR structure by CRISPR technology (Table 3).

**Table 3:** Safety overview of NK cell vaccines in solid tumors.

Safety overview of natural killer (NK) cell vaccines for solid tumors			
Adverse event type	Incidence/severity	Clinical manifestations	Management strategies
Injection site reaction	High (>60%), Grade 1-2	Redness, pain, itching	Local cold compress, NSAIDs for symptomatic relief
Flu-like symptoms	High (50-70%), Grade 1-2	Fever, fatigue, myalgia	Prophylactic antipyretics (acetaminophen), hydration support
Immune-related pneumonitis	Low-Moderate (5-15%), ≥Grade 3 in 3-5%	Cough, dyspnea, radiographic infiltrates	Treatment pause, glucocorticoid therapy (oral or IV)
Thyroid dysfunction	Moderate (10-20%). ≥Grade 3 rare	Hypo/hyperthyroidism symptoms, TSH abnormalities	Endocrinology consult, hormone replacement therapy
Cytokine release syndrome (CRS)	Low(<10%),Severe(2Grade 3) rare	Fever, hypotension, multi-organ dysfunction	Tocilizumab, glucocorticoids for severe cases
Hepatotoxicity	Low-Moderate (5-15%), ≥Grade 3 in 2-4%	Transaminase elevation, bilirubin elevation	Treatment pause, hepatoprotectants (glutathione), glucocorticolds if needed
Off-target toxicity	Low (<5%), severe cases rare	Non-target organ damage(e.g., lung, liver)	Targeting strategy adjustment, CAR optimization via gene editing

## 4 Challenges in clinical translation

Clinical translation of NK cell vaccines in treating solid tumors faces many challenges, which mainly include complex manufacturing processes, ineffective delivery, heterogeneity in patient response, and difficulties in optimization of combination regimens, with an urgent need for breakthroughs through multidisciplinary approaches (57–59).

There are challenges in manufacturing NK cell vaccines. Air pollutants can disrupt the NF-κB pathway, thereby leading to immune dysregulation and immune diseases. Similarly, the influence of environmental factors on the immune system is also reflected in the research on NK cell vaccines. The effectiveness of these vaccines may be regulated by the body’s inflammatory state and the activation of immune pathways, which suggests that when designing and evaluating clinical trials of NK cell vaccines, we need to comprehensively consider the potential impact of environmental factors on the immune system (60). Autologous NK cells have limited expansion capability, long preparation cycle (usually 2–3 weeks), and are costly; Allogeneic sources (for example, umbilical cord blood or IPSC-differentiated NK cells) are suitable for the generation of “off-the-shelf” products, but cell stability and persistence of activity are challenging to maintain (61), whereas functional impairment is likely to occur after *in vitro* expansion (59, 61, 62). Nowadays, standardization of quality control of vaccines has not been achieved; gene editing (for example, CAR-NK or IL-15 autocrine modification) needs to be carried out under good manufacturing practice (GMP) conditions, but there are no consistent standards for homogeneity of cell types, viability after cryopreservation, and recovery, and potency. This hinders large-scale clinical application (62, 63).

There are also many problems with vaccine delivery and tumor targeting. The physical density of the tumor tissue, i.e., fibrotic interstitium, hinders NK cell infiltration; Regulatory T cells (Treg), hypoxia, and metabolites (LysoPS) in the immunosuppressive microenvironment (TME) directly suppress NK cell function through receptor GPR34 (56, 64, 65). If the tumor specificity of the vaccine is not high, it will also harmlessly damage normal tissues, as tumor-associated antigens (TAAs) may also be expressed at low levels in normal tissues. While new designs, e.g., the multi-module DNA nanodevice MODERN, can achieve spatially selective imaging of granzyme A in tumors, targeted delivery *in vivo* systems still need to be optimized (58, 66).

Patient stratification and combination strategy of NK vaccines with other treatments also need to be optimized. No useful predictive markers of efficacy are available currently. Investigation has identified NK cell marker gene signatures in pancreatic cancer (for example, the 7-gene prognostic model) that can be correlated with immune infiltration status, but whether they are clinically universal remains to be validated (54). NK vaccine + immune checkpoint inhibitors (e.g., PD-1 antibodies), oncolytic viruses, or small molecule drugs (HDAC inhibitors) can enhance efficacy, but the timing of administration (sequential or synchronous), modulation of dose (e.g., low-dose PD-1 inhibitors lower costs but require adaptation to cancer type), and mechanism of toxicity superposition are unclear 4,710. GPR34 inhibitors + TIGIT antibodies, for example, are powerfully synergistic in liver cancer models, but data from human trials are limited (57, 64, 67).

## 5 Future prospects

Natural killer (NK) cell vaccines, a novel strategy for immunotherapy of solid tumors, will break the limitations of existing therapies in the future, especially in individualized design, technological innovation of engineering platforms, and perioperative application scenarios. At present, individualized vaccine design based on tumor-specific antigens is an increasing trend. For example, a UK group discovered that the XPO1 protein could be a target of NK cell activation, and its derived peptide, when presented by the HLA-C molecule, could be specifically recognized by the NK cell surface receptor KIR2DS2, demonstrating a novel target for the development of personalized vaccines. These vaccines have the potential to enhance the precision of immune responses in very heterogeneous solid tumors such as liver cancer and head and neck cancer (68–70).

Then, the technology platform for enhancing generality and utility has been developed. The iPSC-NK universal platform facilitates genetic modification to enhance cell functions. For instance, by knocking out inhibitory receptors such as PD-1, the anti-suppression ability of NK cells in the tumor microenvironment can be enhanced; and through genetic engineering, specific receptors such as CAR can be introduced, enabling NK cells to precisely target tumor antigens and improve their killing efficacy (71). At the delivery and function enhancement level, TGFβ inhibition can be countered by non-viral vector antibody conjugating techniques (e.g., IBR854 injection) or gene editing knockout of SMAD4. It can enhance the infiltration and activity of NK cells in the TME, 68NK vaccine delivery capability and activity; Combination use, the combination of NK vaccines with PD-1 inhibitors (e.g., pembrolizumab) synergistic enhancement of anti-tumor immunity achieved an objective response rate of 50% in chemotherapy-resistant biliary tract cancer patients, and the duration of effect lasted for 12–18 months (72).

In addition, clinicians ought to pay attention to the role of NK vaccines in reducing the risk of metastasis after surgery. NK vaccine administration has been utilized postoperatively for the removal of circulating tumor cells, reduction of distant metastasis as well as prevention of recurrence (73). For example, NK vaccine-treated colorectal cancer patients experienced no local recurrence within 3 years (74); NK cells combined with cetuximab for non-small cell lung cancer reduced tumor volume dramatically (up to 70% reduction), which created better conditions for surgery (75); Cryotherapy combined with NK cells for liver cancer enhanced disease control to 85.2% and extended median survival to 9.1 months (76). Furthermore, NK cell vaccines rely on the recognition and presentation mechanisms of tumor antigens. Clinicians have used the IP scoring system to evaluate the potential efficacy of NK cell vaccines in different patient groups, providing new biomarker references that can help identify patients more likely to benefit from NK cell vaccine therapy, thereby optimizing clinical trial design and treatment strategies (77).

NK cell vaccines will revolutionize the treatment of solid tumors in three ways: innovation targeting, upgrading engineering, and expansion of clinical application scenarios, especially with the possibility of becoming a “standard therapy” in perioperative comprehensive treatment, to provide more convenient and effective immune protection for patients.

## 6 Conclusion

Natural killer (NK) cell vaccines, as a new generation of immunotherapy for solid tumors, have shown great potential to break through the bottleneck of existing treatments by reshaping the tumor microenvironment, overcoming antigen presentation barriers, and targeting MHC-I low-expressing tumors. A large number of ongoing Phase I/II/III clinical trials (e.g., DC vaccines, mRNA/LNP platforms, and NK receptor agonist antibodies) constitute the key driving force for the development of this field. Early evidence shows 30–50% objective response rates and >80% disease control rates for some therapies. Sophisticated manufacturing processes, inefficient delivery, and an immunosuppressive microenvironment remain the major translational challenges. There is going to be a need in the future to expand interdisciplinary collaboration (basic research optimizing target screening, engineering technology developing carrier design, clinical medical confirmation integrating strategy, and regulatory science standardizing) to facilitate clinical translation. Guarded optimistic expectations are that seminal events can occur in the next 2–3 years: Such as Phase II survival benefit data for HER2/EGFR-targeting bispecific antibodies (e.g., AFM24), Phase III data for a universal iPSC-NK vaccine combined with a PD-1 inhibitor, and conditional approval for the first solid tumor indication (e.g., glioblastoma DC vaccine).

## References

[1] Fan Z Deng J Wang Y Fan X Xie J. Bladder cancer: immunotherapy and pelvic lymph node dissection. Vaccines (Basel). (2024) 12:150. doi: 10.3390/vaccines12020150, PMID: 38400134 PMC10893107

[2] Hawke LG Mitchell BZ Ormiston ML. TGF-β and IL-15 synergize through MAPK pathways to drive the conversion of human NK cells to an innate lymphoid cell 1-like phenotype. J Immunol. (2020) 204:3171–81. doi: 10.4049/jimmunol.1900866, PMID: 32332109

[3] Zhou Y Cheng L Liu L Li X. NK cells are never alone: crosstalk and communication in tumour microenvironments. Mol Cancer. (2023) 22:34. doi: 10.1186/s12943-023-01737-7, PMID: 36797782 PMC9933398

[4] Navin I Lam MT Parihar R. Design and implementation of NK cell-based immunotherapy to overcome the solid tumor microenvironment. Cancers (Basel). (2020) 12:3871. doi: 10.3390/cancers12123871, PMID: 33371456 PMC7767468

[5] Wolf NK Kissiov DU Raulet DH. Roles of natural killer cells in immunity to Cancer, and applications to immunotherapy. Nat Rev Immunol. (2023) 23:90–105. doi: 10.1038/s41577-022-00732-1 35637393

[6] Jianfeng W Yutao W Jianbin B. Long non-coding RNAs correlate with genomic stability in prostate cancer: a clinical outcome and survival analysis. Genomics. (2021) 113:3141–51. doi: 10.1016/j.ygeno.2021.06.029 34174340

[7] Yan K Wang Y Shao Y Xiao T. Gene instability-related lncRNA prognostic model of melanoma patients via machine learning strategy. J Oncol. (2021) 2021:5582920. doi: 10.1155/2021/5582920, PMID: 34122546 PMC8169244

[8] Wang Y Yan K Lin J Li J Bi J. Macrophage M2 co-expression factors correlate with the immune microenvironment and predict outcome of renal clear cell carcinoma. Front Genet. (2021) 12:615655. doi: 10.3389/fgene.2021.615655 33692827 PMC7938896

[9] Wang Y Yan K Wang J Lin J Bi J. Macrophage co-expression factors correlate with immune phenotype and predict prognosis of bladder Cancer. Front Oncol. (2021) 11:609334. doi: 10.3389/fonc.2021.609334 33828973 PMC8019942

[10] Wang Y Yan K Lin J Liu Y Wang J Li X. CD8+ T cell co-expressed genes correlate with clinical phenotype and microenvironments of urothelial cancer. Front Oncol. (2020) 10:553399. doi: 10.3389/fonc.2020.553399, PMID: 33330025 PMC7713665

[11] Zhao X Xiong J Li D Zhang Y. Clinical trials of nanoparticle-enhanced CAR-T and NK cell therapies in oncology: Overcoming Translational and Clinical Challenges -a Mini Review. Front Med. (2025) 12:1655693. doi: 10.3389/fmed.2025.1655693 PMC12403998 40904365

[12] Kang X Li D Sun R. Nanotechnology and natural killer cell immunotherapy: synergistic approaches for precise immune system adjustment and targeted cancer treatment in gastrointestinal tumors. Front Med. (2025) 12:1647737. doi: 10.3389/fmed.2025.1647737 PMC12408553 40917849

[13] Zhu T Li Y Wang Y Li D. The application of dendritic cells vaccines in tumor therapy and their combination with biomimetic nanoparticles. Vaccines (Basel). (2025) 13:337. doi: 10.3390/vaccines13040337, PMID: 40333202 PMC12031636

[14] Wculek SK Cueto FJ Mujal AM Melero I Krummel MF Sancho D. Dendritic cells in Cancer immunology and immunotherapy. Nat Rev Immunol. (2020) 20:7–24. doi: 10.1038/s41577-019-0210-z 31467405

[15] Fotaki G Jin C Kerzeli IK Ramachandran M Martikainen M-M Karlsson-Parra A. Cancer vaccine based on a combination of an infection-enhanced adenoviral vector and pro-inflammatory allogeneic DCs leads to sustained antigen-specific immune responses in three melanoma models. Onco Targets Ther. (2017) 7:e1397250. doi: 10.1080/2162402X.2017.1397250, PMID: 29399398 PMC5790347

[16] Fotaki G Jin C Ramachandran M Kerzeli IK Karlsson-Parra A Yu D. Pro-inflammatory allogeneic DCs promote activation of bystander immune cells and thereby license antigen-specific T-cell responses. Onco Targets Ther. (2017) 7:e1395126. doi: 10.1080/2162402X.2017.1395126 PMC5790348 29399392

[17] Karlsson-Parra A Kovacka J Heimann E Jorvid M Zeilemaker S Longhurst S. Ilixadencel - an allogeneic cell-based anticancer immune primer for intratumoral administration. Pharm Res. (2018) 35:156. doi: 10.1007/s11095-018-2438-x, PMID: 29904904 PMC6002422

[18] Lindskog M Laurell A Kjellman A Melichar B Rey PM Zieliński H. Ilixadencel, a cell-based immune primer, plus sunitinib versus sunitinib alone in metastatic renal cell carcinoma: a randomized phase 2 study. Eur Urol Open Sci. (2022) 40:38–45. doi: 10.1016/j.euros.2022.03.012, PMID: 35638086 PMC9142735

[19] Preusser M van den Bent MJ. Autologous tumor lysate-loaded dendritic cell vaccination (DCVax-L) in glioblastoma: breakthrough or fata morgana? Neuro-oncol. (2023) 25:631–4. doi: 10.1093/neuonc/noac281 36562460 PMC10076936

[20] Bakhtiyaridovvombaygi M Yazdanparast S Mikanik F Izadpanah A Parkhideh S Shahbaz ghasabeh A. Cytokine-induced memory-like NK cells: emerging strategy for AML immunotherapy. Biomed Pharmacother. (2023) 168:115718. doi: 10.1016/j.biopha.2023.115718, PMID: 37857247

[21] Romee R Cooley S Berrien-Elliott MM Westervelt P Verneris MR Wagner JE. First-in-human phase 1 clinical study of the IL-15 superagonist complex ALT-803 to treat relapse after transplantation. Blood. (2018) 131:2515–27. doi: 10.1182/blood-2017-12-823757, PMID: 29463563 PMC5992862

[22] Safran H Cassier PA Vicier C Forget F Gomez-Roca CA Penel N. Phase 1/2 study of DF1001, a novel tri-specific, NK cell engager therapy targeting HER2, in patients with advanced solid tumors: phase 1 DF1001 monotherapy dose-escalation results. JCO. (2023) 41:2508–8. doi: 10.1200/JCO.2023.41.16_suppl.2508

[23] El-Khoueiry A Saavedra O Thomas J Livings C Garralda E Hintzen G. First-in-human phase I study of a CD16A bispecific innate cell engager, AFM24, targeting EGFR-expressing solid tumors. Clin Cancer Res. (2025) 31:1257–67. doi: 10.1158/1078-0432.CCR-24-1991 39846810 PMC11964176

[24] Jongen-Lavrencic M Garciaz S Huls GA Maiti A Boissel N Botton SD. A first-in-human study of CD123 NK cell engager SAR443579 in relapsed or refractory acute myeloid leukemia, B-cell acute lymphoblastic leukemia, or high-risk myelodysplasia. J Clin Oncol. (2023). 41:7005. doi: 10.1200/JCO.2023.41.16_suppl.7005

[25] Delehedde C Ciganek I Bernard PL Laroui N Da Silva CC Gonçalves C. Enhancing natural killer cells proliferation and cytotoxicity using imidazole-based lipid nanoparticles encapsulating Interleukin-2 mRNA. Mol Ther Nucleic Acids. (2024) 35:102263. doi: 10.1016/j.omtn.2024.102263 39104868 PMC11298638

[26] Li F Zhang X-Q Ho W Tang M Li Z Bu L. mRNA lipid nanoparticle-mediated pyroptosis sensitizes immunologically cold tumors to checkpoint immunotherapy. Nat Commun. (2023) 14:4223. doi: 10.1038/s41467-023-39938-9, PMID: 37454146 PMC10349854

[27] Zhong R Ling X Cao S Xu J Zhang B Zhang X. Safety and efficacy of dendritic cell-based immunotherapy (DCVAC/LuCa) combined with carboplatin/pemetrexed for patients with advanced non-squamous non-small-cell lung cancer without oncogenic drivers. ESMO Open. (2022) 7:100334. doi: 10.1016/j.esmoop.2021.100334, PMID: 34959168 PMC8718955

[28] Atmaca A Alt J Baz DV Dziadziuszko R Moreno V Pose V. 1486 preliminary results from LuCa-MERIT-1, a phase I trial evaluating BNT116, a fixed antigen mRNA vaccine, plus Cemiplimab in advanced non-small cell lung Cancer after progression on PD-1 inhibition In: Proceedings of the Late-Breaking: BMJ Publishing Group Ltd (2024). A1716–6.

[29] Tosch C Bastien B Barraud L Grellier B Nourtier V Gantzer M. Viral based vaccine TG4010 induces broadening of specific immune response and improves outcome in advanced NSCLC. J Immunother Cancer. (2017) 5:70. doi: 10.1186/s40425-017-0274-x, PMID: 28923084 PMC5604422

[30] Urrizola A Solans B Bayona RS Mejías L Vilalta A Cruz SDL. 1029P addition of dendritic cell vaccines to Neoadjuvant chemotherapy in HER2 negative breast Cancer patients. Ann Oncol. (2020) 31:S710. doi: 10.1016/j.annonc.2020.08.1149 PMC8721381 34987618

[31] Berneman ZN De Laere M Germonpré P Huizing MT Willemen Y Lion E. WT1-mRNA dendritic cell vaccination of patients with glioblastoma multiforme, malignant pleural mesothelioma, metastatic breast cancer, and other solid tumors: type 1 T-lymphocyte responses are associated with clinical outcome. J Hematol Oncol. (2025) 18. doi: 10.1186/s13045-025-01661-x PMC11755790 39849594

[32] Li B Zhu X Ge J Zhang H Gao Y Bao X. Intraperitoneal infusion of NKG2D CAR-NK cells induces endogenous CD8+ T cell activation in patients with advanced colorectal Cancer. Mol Ther. (2025). doi: 10.1016/j.ymthe.2025.05.026 PMC12432867 40437757

[33] Yuan Y Egelston C Colunga Flores O Chaurasiya S Lin D Chang H. CF33-hNIS-anti-PD-L1 oncolytic virus followed by trastuzumab-deruxtecan in a patient with metastatic triple negative breast cancer: a case study. Ther Adv Med Oncol. (2023) 15:17588359231210675. doi: 10.1177/17588359231210675, PMID: 38028143 PMC10640805

[34] Xiao S Chen X Zu X. Advancements in immunotherapy research for colorectal cancer based on microsatellite status. Sci Sin-Vitae. (2023) 53:1455–66. doi: 10.1360/SSV-2023-0103

[35] 卵巢癌新辅助树突状细胞疫苗接种 | 临床研究试验登记(上皮性卵巢癌 | 卵巢癌)(NCT05773859) --- NEOadjuvant dendritic cell vaccination for ovarian Cancer | clinical research trial listing (epithelial ovarian Cancer | ovarian carcinoma) (NCT05773859). (2025). Available online at: https://www.trialx.com/clinical-trials/listings/234423/neoadjuvant-dendritic-cell-vaccination-for-ovarian-cancer/ (Accessed June 22, 2025).

[36] Thaker PH Richardson DL Hagemann AR Holloway RW Reed M Bergman MK. OVATION-2: a randomized phase I/II study evaluating the safety and efficacy of IMNN-001 (IL-12 gene therapy) with neo/adjuvant chemotherapy in patients newly-diagnosed with advanced epithelial ovarian cancer. Gynecol Oncol. (2025) 197:182–91. doi: 10.1016/j.ygyno.2025.04.578, PMID: 40461366

[37] Dorigo O Oza AM Pejovic T Ghatage P Ghamande S Provencher D. Maveropepimut-S, a DPX-based immune-educating therapy, shows promising and durable clinical benefit in patients with recurrent ovarian Cancer, a phase II trial. Clin Cancer Res. (2023) 29:2808–15. doi: 10.1158/1078-0432.CCR-22-2595 37126016 PMC10390884

[38] Gogineni V Morand S Staats H Royfman R Devanaboyina M Einloth K. Current ovarian cancer maintenance strategies and promising new developments. J Cancer. (2021) 12:38–53. doi: 10.7150/jca.49406, PMID: 33391401 PMC7738841

[39] Morimoto T Nakazawa T Maeoka R Nakagawa I Tsujimura T Matsuda R. Natural killer cell-based immunotherapy against glioblastoma. Int J Mol Sci. (2023) 24:2111. doi: 10.3390/ijms24032111, PMID: 36768432 PMC9916747

[40] Agosti E Zeppieri M De Maria L Tedeschi C Fontanella MM Panciani PP. Glioblastoma immunotherapy: a systematic review of the present strategies and prospects for advancements. Int J Mol Sci. (2023) 24:15037. doi: 10.3390/ijms242015037, PMID: 37894718 PMC10606063

[41] Perales R Perales-Puchalt A Bartha G Northcott J Chen R Lyle J. 692 circulating tumor DNA analysis of advanced hepatocellular cancer (HCC) patients treated with neoantigen targeted personalized cancer DNA vaccine (GNOS-PV02) in combination with plasmid IL-12 (pIL12) and anti-PD1 (pembrolizumab). J Immunother Cancer. (2022) 10:692. doi: 10.1136/jitc-2022-SITC2022.0692

[42] Lian G Mak TS-K Yu X Lan H-Y. Challenges and recent advances in NK cell-targeted immunotherapies in solid tumors. Int J Mol Sci. (2021) 23:164. doi: 10.3390/ijms23010164, PMID: 35008589 PMC8745474

[43] Bai R Cui J. Burgeoning exploration of the role of natural killer cells in anti-PD-1/PD-L1 therapy. Front Immunol. (2022):13. doi: 10.3389/fimmu.2022.886931 PMC9133458 35634343

[44] Jiang S Guo H Qu H Bai Y Shi B Zhang P. 9MW2821, a novel Nectin-4 antibody-drug conjugate (ADC), combined with Toripalimab in treatment-naïve patients with locally advanced or metastatic urothelial carcinoma (La/mUC): results from a phase 1b/2 study. J Clin Oncol. (2025) 43:4519. doi: 10.1200/JCO.2025.43.16_suppl.4519

[45] Bartlett NL Herrera AF Domingo-Domenech E Mehta A Forero-Torres A Garcia-Sanz R. A phase 1b study of AFM13 in combination with pembrolizumab in patients with relapsed or refractory Hodgkin lymphoma. Blood. (2020) 136:2401–9. doi: 10.1182/blood.2019004701, PMID: 32730586 PMC7685206

[46] Baugh R Khalique H Page E Lei-Rossmann J Wan PK-T Johanssen T. Targeting NKG2D ligands in glioblastoma with a bispecific T-cell engager is augmented with conventional therapy and enhances oncolytic Virotherapy of glioma stem-like cells. J Immunother Cancer. (2024) 12:e008460. doi: 10.1136/jitc-2023-008460 38724464 PMC11086472

[47] Weiss T Schneider H Silginer M Steinle A Pruschy M Polić B. NKG2D-dependent antitumor effects of chemotherapy and radiotherapy against glioblastoma. Clin Cancer Res. (2018) 24:882–95. doi: 10.1158/1078-0432.CCR-17-1766, PMID: 29162646

[48] Choi MG Son GW Choi MY Jung JS Rho JK Ji W. Safety and efficacy of SNK01 (autologous natural killer cells) in combination with cytotoxic chemotherapy and/or cetuximab after failure of prior tyrosine kinase inhibitor in non-small cell lung cancer: non-clinical mouse model and phase I/IIa clinical study. J Immunother Cancer. (2024) 12:e008585. doi: 10.1136/jitc-2023-008585, PMID: 38538093 PMC10982808

[49] Nieto Y Banerjee P Kaur I Basar R Li Y Daher M. Allogeneic NK cells with a bispecific innate cell engager in refractory relapsed lymphoma: a phase 1 trial. Nat Med. (2025) 31:1987–93. doi: 10.1038/s41591-025-03640-8, PMID: 40186077 PMC13218020

[50] Kiefer A Prüfer M Röder J Pfeifer Serrahima J Bodden M Kühnel I. Dual targeting of glioblastoma cells with bispecific killer cell engagers directed to EGFR and ErbB2 (HER2) facilitates effective elimination by NKG2D-CAR-engineered NK cells. Cells. (2024) 13:246. doi: 10.3390/cells13030246, PMID: 38334638 PMC10854564

[51] van t Land FR Willemsen M Bezemer K van der Burg SH van den Bosch TPP Doukas M. Dendritic Cell–Based Immunotherapy in Patients with Resected Pancreatic Cancer. J Clin Oncol. (2024) 42:3083–93. doi: 10.1200/JCO.23.02585 38950309 PMC11379361

[52] Aspirin AP de Los Reyes V AA Kim Y. Polytherapeutic strategies with oncolytic virus-bortezomib and adjuvant NK cells in cancer treatment. J R Soc Interface. (2021) 18:20200669. doi: 10.1098/rsif.2020.0669, PMID: 33402021 PMC7879760

[53] Tambaro P Mahadeo KM Khazal SJ Tewari P Petropoulos D Slopis JM. Immune effector cell associated neurotoxicity (ICANS) among pediatric and AYA patients: MD Anderson Cancer Center Experience免疫效应细胞相关神经毒性(ICANS)在儿童和青年成人患者中的表现:MD 安德森癌症中心经验. Biol Blood Marrow Transplant. (2020) 26:S316. doi: 10.1016/j.bbmt.2019.12.397

[54] Yan J Zhang C Xu Y Huang Z Ye Q Qian X. Clinical efficacy analysis of 23 advanced solid tumor patients who developed immune-related adverse events during immunotherapy. Chin. J. Microbiol. Immunol. (2020) 40:382–386, doi: 10.3760/cma.j.cn112309-20200325-00144

[55] Hadjis AD McCurdy SR. The role and novel use of natural killer cells in graft-versus-leukemia reactions after allogeneic transplantation. Front Immunol. (2024) 15:1358668. doi: 10.3389/fimmu.2024.1358668, PMID: 38817602 PMC11137201

[56] Bagus BI. Autologous natural killer cells as a promising immunotherapy for locally advanced colon adenocarcinoma: three years follow-up of resectable case. Cancer Rep. (2023) 6:e1866. doi: 10.1002/cnr2.1866, PMID: 37439389 PMC10480413

[57] Yan J Zhang C Xu Y Huang Z Ye Q Qian X. GPR34 is a metabolic immune checkpoint for ILC1-mediated antitumor immunity. Nat Immunol. (2024) 25:2057–67. doi: 10.1038/s41590-024-01973-z 39358444

[58] Vivier E Rebuffet L Narni-Mancinelli E Cornen S Igarashi RY Fantin VR. Natural killer cell therapies. Nature. (2024) 626:727–36. doi: 10.1038/s41586-023-06945-1 38383621

[59] Wang K Wang L Wang Y Xiao L Wei J Hu Y. Reprogramming natural killer cells for Cancer therapy. Mol Ther. (2024) 32:2835–55. doi: 10.1016/j.ymthe.2024.01.027 38273655 PMC11403237

[60] Cui C Yang R Chen H Li D Sun X Wang Y. Air toxins disorder the NF-kB pathway leads to immune disorders and immune diseases in the human health. Ecotoxicol Environ Saf. (2025) 302:118474. doi: 10.1016/j.ecoenv.2025.118474 40532606

[61] Goldenson BH Hor P Kaufman DS. iPSC-derived natural killer cell therapies - expansion and targeting. Front Immunol. (2022) 13:841107. doi: 10.3389/fimmu.2022.841107, PMID: 35185932 PMC8851389

[62] Caporale JR Naeimi Kararoudi M Lamb MG Lee DA. Dark NKnight rising: a current perspective on NK cell and CAR-NK cell therapy. Cytotherapy. (2025) 27:812–25. doi: 10.1016/j.jcyt.2025.04.064 40459488

[63] Xue D Lu S Zhang H Zhang L Dai Z Kaufman DS. Induced pluripotent stem cell-derived engineered T cells, natural killer cells, macrophages, and dendritic cells in immunotherapy. Trends Biotechnol. (2023) 41:907–22. doi: 10.1016/j.tibtech.2023.02.003, PMID: 36858941

[64] Jiménez-Labaig P Mohamed F Tan NJI Sanna I El Bairi K Khan SZ. Expanding access to cancer immunotherapy: a systematic review of low-dose PD-(L)1 inhibitor strategies. Eur J Cancer. (2025) 225:115564. doi: 10.1016/j.ejca.2025.115564, PMID: 40513286

[65] Devillier R Chrétien A-S Pagliardini T Salem N Blaise D Olive D. Mechanisms of NK cell dysfunction in the tumor microenvironment and current clinical approaches to harness NK cell potential for immunotherapy. J Leukoc Biol. (2021) 109:1071–88. doi: 10.1002/JLB.5MR0920-198RR, PMID: 32991746

[66] Engineering of a multi-modular DNA Nanodevice for Spatioselective imaging and evaluation of NK cell-mediated Cancer immunotherapy - Chen - 2025 - Angewandte Chemie international edition - Wiley online library. (2025). Available online at: https://onlinelibrary.wiley.com/doi/10.1002/anie.202414064 (Accessed June 28, 2025). 10.1002/anie.202414064 39375853

[67] Shaik R Chittepu SM Tarapatla M Begum F Vempati S Royyala A. Chemoimmunotherapy synergism: mechanisms and clinical applications. Naunyn Schmiedeberg's Arch Pharmacol. (2025). doi: 10.1007/s00210-025-04125-8 40220027

[68] Azmi AS Uddin MH Mohammad RM. The nuclear export protein XPO1 - from biology to targeted therapy. Nat Rev Clin Oncol. (2021) 18:152–69. doi: 10.1038/s41571-020-00442-4 33173198

[69] O’Hara MP Yanamandra AV Sastry KJ. Immunity from NK cell subsets is important for vaccine-mediated protection in HPV+ cancers. Vaccines (Basel). (2024) 12:206. doi: 10.3390/vaccines12020206 38400189 PMC10892709

[70] Afolabi LO Adeshakin AO Sani MM Bi J Wan X. Genetic reprogramming for NK cell cancer immunotherapy with CRISPR/Cas9. Immunology. (2019) 158:63–9. doi: 10.1111/imm.13094, PMID: 31315144 PMC6742769

[71] Wei X Su C Liu Y Wei N Xiang K Qian Q. IPSC-derived NK cells for immunotherapy and therapeutic perspective (review). Mol Med Rep. (2025) 32:222. doi: 10.3892/mmr.2025.13587 40476558 PMC12174902

[72] Leem G Jang S-I Cho J-H Jo JH Lee HS Chung MJ. Safety and efficacy of allogeneic natural killer cells in combination with Pembrolizumab in patients with chemotherapy-refractory biliary tract Cancer: a multicenter open-label phase 1/2a trial. Cancers (Basel). (2022) 14:4229. doi: 10.3390/cancers14174229 36077766 PMC9454779

[73] Park CG Hartl CA Schmid D Carmona EM Kim H-J Goldberg MS. Extended release of perioperative immunotherapy prevents tumor recurrence and eliminates metastases. Sci Transl Med. (2018) 10:eaar1916. doi: 10.1126/scitranslmed.aar1916 29563317

[74] Vyas M Requesens M Nguyen TH Peigney D Azin M Demehri S. Natural killer cells suppress Cancer metastasis by eliminating circulating Cancer cells. Front Immunol. (2022) 13:1098445. doi: 10.3389/fimmu.2022.1098445 36733396 PMC9887278

[75] Liang S Lin M Niu L Xu K Wang X Liang Y. Cetuximab combined with natural killer cells therapy: an alternative to Chemoradiotherapy for patients with advanced non-small cell lung Cancer (NSCLC). Am J Cancer Res. (2018) 8:879–91. doi: 10.7150/jacr.24744 29888109 PMC5992505

[76] Lin M Liang S Wang X Liang Y Zhang M Chen J. Cryoablation combined with allogenic natural killer cell immunotherapy improves the curative effect in patients with advanced hepatocellular Cancer. Oncotarget. (2017) 8:81967–77. doi: 10.18632/oncotarget.17804 29137237 PMC5669863

[77] Wang Y Yan K Guo Y Lu Y Su H Li H. IP-score correlated to endogenous tumour antigen peptide processing: a candidate clinical response score algorithm of immune checkpoint inhibitors therapy in multiple cohorts. Front Immunol. (2023) 13:1085491. doi: 10.3389/fimmu.2022.1085491, PMID: 36700205 PMC9868931

